# Early Multivessel Stent Thrombosis Causing Rapid Progression From Non-ST-Segment Elevation Myocardial Infarction (NSTEMI) to ST-Segment Elevation Myocardial Infarction (STEMI) Requiring Mechanical Circulatory Support

**DOI:** 10.7759/cureus.105366

**Published:** 2026-03-17

**Authors:** Mafaz Mansoor, Asma Yasin, Patrick Joseph, Roberta Baah-Sackey

**Affiliations:** 1 Internal Medicine, South Georgia Medical Center, Valdosta, USA

**Keywords:** drug-eluting stents, early stent thrombosis, intravascular ultrasound (ivus), mechanical circulatory support, multivessel thrombosis, percutaneous coronary intervention (pci)

## Abstract

Early stent thrombosis is an uncommon but life-threatening complication of percutaneous coronary intervention (PCI), associated with high mortality despite adherence to dual antiplatelet therapy (DAPT). Early stent thrombosis is a rare but serious complication of percutaneous coronary intervention that is often associated with significant morbidity and mortality. Multifactorial contributors, including diabetes mellitus and reduced left ventricular ejection fraction, significantly increase the risk. We present the case of a 50-year-old African American male with no prior medical history, presenting with persistent chest pain, nausea, vomiting, and diaphoresis. Initial workup revealed elevated troponins, and coronary angiography identified critical stenoses in the left anterior descending (LAD) and obtuse marginal (OM) arteries, which were treated with PCI with drug-eluting stents followed by DAPT. Further investigation revealed new-onset heart failure, diabetes, and hypercholesterolemia, prompting initiation of guideline-directed medical therapy (GDMT), insulin, and atorvastatin, respectively. The patient was discharged but returned within 24 hours with the new onset of chest pain. Emergency coronary angiography demonstrated occlusion of the proximal LAD at the stented segment as well as thrombotic occlusion involving the circumflex/OM stent, consistent with early stent thrombosis. Intravascular ultrasound (IVUS) performed during the repeat intervention demonstrated a relatively large reference vessel diameter of approximately 4.5 mm with areas of suboptimal stent expansion that may have contributed to thrombus formation. IVUS-guided repeat PCI was performed with high-pressure balloon post-dilation and additional stent optimization to restore thrombolysis in myocardial infarction grade 3 (TIMI-3) flow. Due to significant hemodynamic compromise with elevated left ventricular end-diastolic pressure (LVEDP) of 40 mmHg and hypoxia requiring non-invasive ventilatory support, mechanical circulatory support with an Impella CP^®^ device was initiated. Following stabilization, the Impella CP^®^ device was removed, GDMT was resumed, blood glucose was optimized, and the patient was discharged home on DAPT after successful management and cardiology clearance with the instruction for close follow-up with cardiology and the primary care physician. This case underscores the complex interplay of patient-related and procedural factors in early stent thrombosis and highlights the critical role of individualized pharmacotherapy, meticulous stent optimization with intravascular imaging, and timely mechanical support in achieving favorable outcomes in high-risk patients.

## Introduction

Acute coronary syndrome (ACS) is a leading cause of morbidity and mortality worldwide [1]. Although management and therapeutic strategies have significantly improved, early recurrence of ACS remains a major challenge, especially when associated with stent thrombosis [2]. Stent thrombosis is classified by timing after stent implantation: early (≤30 days), late (31 days-1 year), and very late (>1 year). Early stent thrombosis is further categorized into acute (within 24 hours) and subacute (1-30 days) after the procedure [3].

Dual antiplatelet therapy (DAPT) is standard following percutaneous coronary intervention (PCI); however, recurrent ischemic events may occur despite adherence, indicating the multifactorial nature of stent thrombosis [4]. Associated risk factors include inadequate dual antiplatelet therapy, stent under-expansion/malposition, diabetes mellitus, renal dysfunction, and reduced ejection fraction [5].

We report a case of a middle-aged man who presented with non-ST-segment elevation myocardial infarction (NSTEMI) and underwent PCI, complicated with a new diagnosis of heart failure and diabetes. Within 24 hours, he re-presented with ST-segment elevation myocardial infarction (STEMI) due to early stent thrombosis of multiple vessels, requiring both revascularization and mechanical circulatory support with an Impella CP^®^ device.

## Case presentation

A 50-year-old African American male with no known past medical history presented with persistent substernal chest pain associated with nausea, vomiting, and diaphoresis for one day. Initial workup at an outside facility revealed elevated troponins, and the patient was transferred to our institution for further evaluation and management. On arrival at our institute, the patient continued to experience active chest pain. Vital signs at presentation were as follows: blood pressure 159/96 mmHg, heart rate 112 beats per minute, oxygen saturation 93% on room air, and temperature 99.3°F. Laboratory investigations are summarized in Table 1. Troponin I on presentation was elevated at 3,631 ng/L and subsequently peaked at >26,000 ng/L. Chest radiography demonstrated no acute cardiopulmonary findings. 

**Table 1:** Laboratory investigations WBC - white blood cell; CK - creatine kinase; aPTT - activated partial thromboplastin time; INR - international normalized ratio; HDL - high density lipoprotein; LDL - low density lipoprotein

Parameter	Result	Reference range
WBC	13,800 /uL	4,800-10,080 /uL
Hemoglobin	15.9 g/dL	14.0-18.0 g/dL
Platelets	194,000 /uL	130,000-400,000 /uL
Troponin I	3,631 ng/L	<19.8 ng/L
CK	1,365 U/L	38-259 U/L
CK-MB	150 ng/mL	0.1-10.0 ng/mL
Myoglobin	93 ng/ml	17-106 ng/ml
Lactate	2.80 mmol/L	0.50-2.00 mmol/L
Activated clotting time	279 seconds	89-137 seconds
aPTT	188 seconds	25.1-36.5 seconds
Prothrombin time	11.6 seconds	9.4-12.5 seconds
INR	1.00	0.8-1.1
Blood glucose	264 mg/dL	68-114 mg/dL
HbA1C	9.9 %	4.0-5.6 %
Cholesterol	140 mg/dL	130-200 mg/dL
Triglycerides	112 mg/dL	≤150 mg/dL
HDL	33.0 mg/dL	≥40.0 mg/dL
LDL cholesterol-calculated	85 mg/dL	0-100 mg/dL

**Figure 1:**
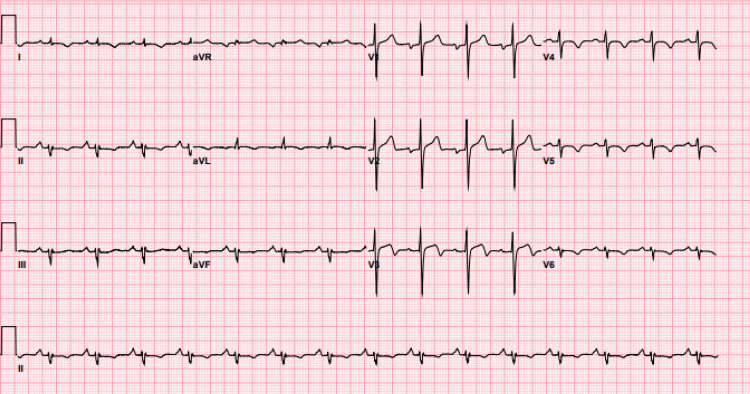
Electrocardiogram on initial presentation At the time of initial presentation, the electrocardiography demonstrated abnormal T-waves in anterolateral leads (Wellen's sign), suggestive of critical proximal left anterior descending (LAD) disease.

Electrocardiogram (ECG) demonstrated sinus tachycardia, left axis deviation, and biphasic T waves in leads V2 and V3 consistent with Wellen's pattern (Figure 1).

**Figure 2:**
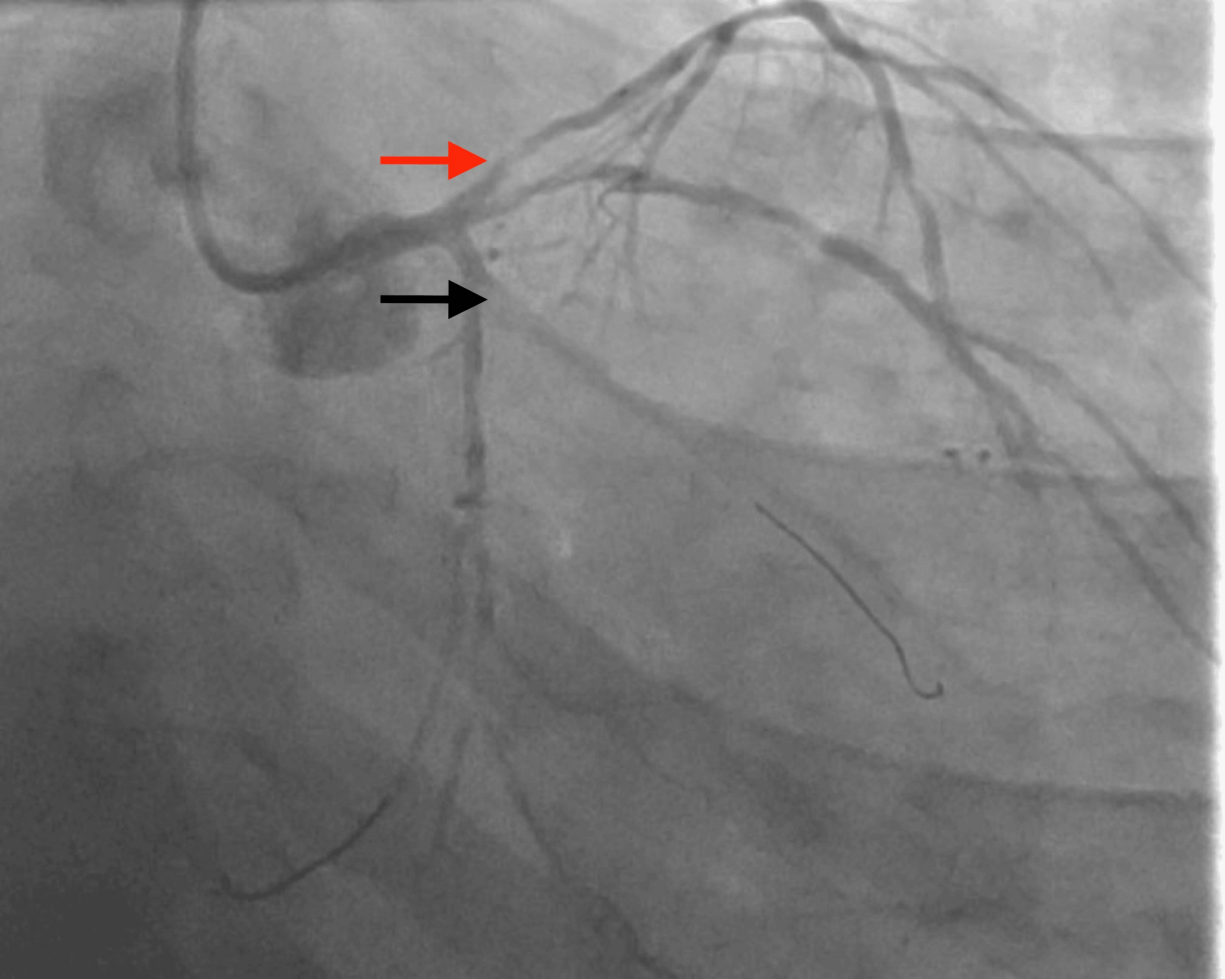
Left heart catheterization on initial presentation Left coronary angiography demonstrating significant obstructive coronary artery disease involving the left anterior descending (LAD) artery (red arrow) and the obtuse marginal (OM) branch of the left circumflex artery (black arrow). Severe stenosis is noted in the proximal segment of the LAD and in the OM branch, resulting in reduced distal coronary flow.

**Figure 3:**
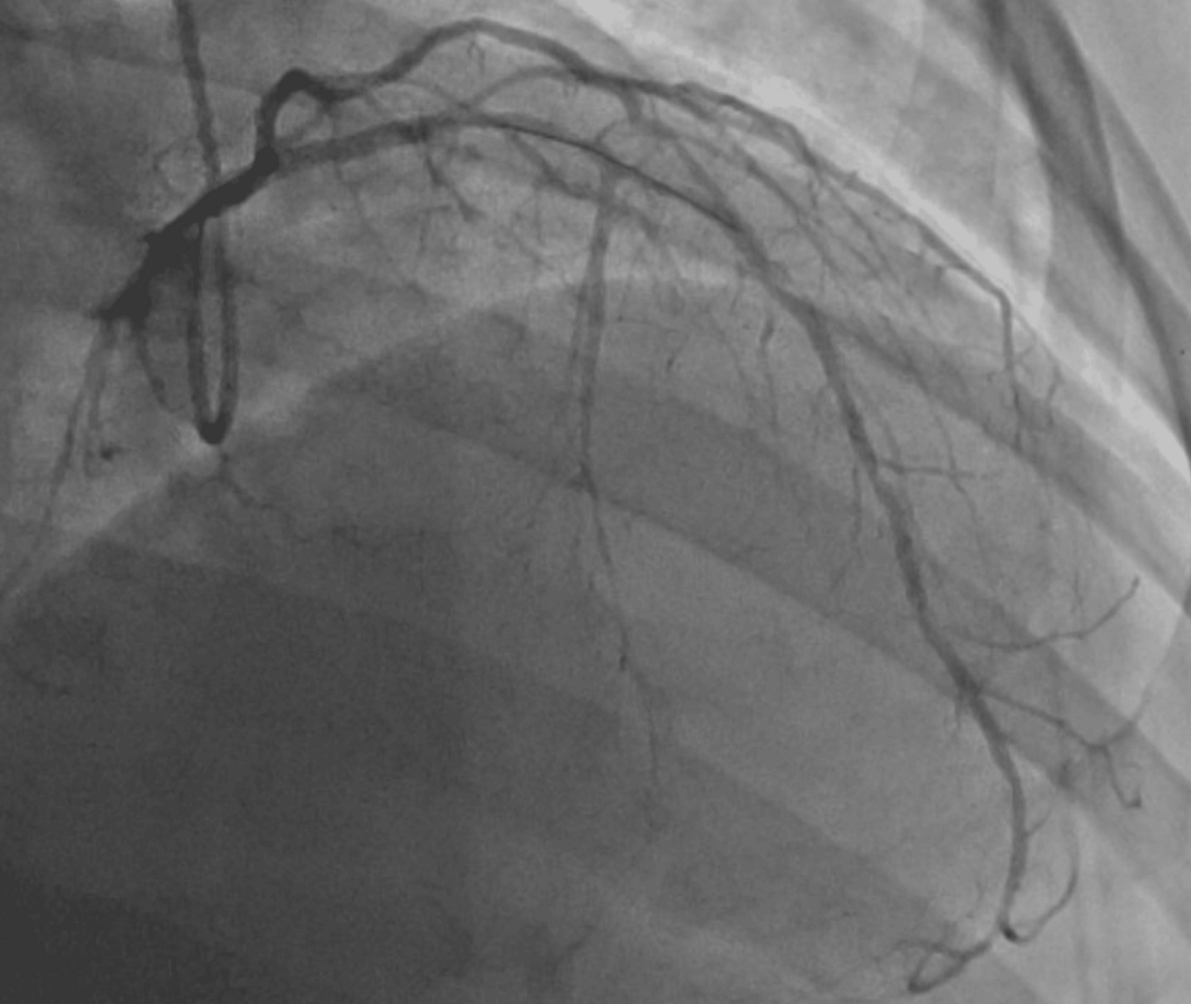
Left heart catheterization Coronary angiography following successful percutaneous coronary intervention with drug-eluting stent placement in the proximal left anterior descending artery and the obtuse marginal branch. The post-intervention images demonstrate restoration of luminal patency with improved distal coronary flow and no evidence of residual significant stenosis.

Prior to arrival at our facility, the patient was already started on intravenous unfractionated heparin infusion following an initial weight-based bolus (approximately 60 units/kg) with a continuous infusion of 12 units/kg/hour, along with an intravenous nitroglycerin infusion initiated at 5 µg/min. The patient had also received aspirin (325 mg) prior to transfer. At the time of presentation to our institution, the patient remained on continuous heparin and nitroglycerin infusions. Cardiology was immediately consulted, and the patient was taken to the cardiac catheterization laboratory within approximately two hours of presentation for urgent coronary angiography. He subsequently underwent PCI, which showed critical 90% proximal left anterior descending (LAD) and 95% obtuse marginal (OM1) stenosis (Figure 2), both treated with Xience Skypoint™ drug-eluting stents (3.0×18 mm in LAD; 2.25×38 mm in OM1) (Figure 3). The right coronary artery (RCA) was the dominant vessel with diffuse mild non-obstructive disease without significant stenosis or collateral circulation to the LAD. Final angiography demonstrated excellent stent expansion and thrombolysis in myocardial infarction grade 3 (TIMI-3) flow without dissection, perforation, or distal embolization. The patient was started on dual antiplatelet therapy (aspirin 81 mg and clopidogrel 75 mg) and atorvastatin 40 mg daily.

During admission, further workup revealed new-onset heart failure with reduced ejection fraction (HFrEF), with a transthoracic echocardiogram (TTE) showing a left ventricular ejection fraction (LVEF) of 30-35%, suspected mid, distal, and apical akinesis, and moderate mitral regurgitation. The patient was also diagnosed with new-onset type 2 diabetes mellitus (glycated hemoglobin, A1c 9.9%). Guideline-directed medical therapy was initiated, including valsartan 40 mg daily, spironolactone 12.5 mg daily, metoprolol 25 mg twice daily, and dapagliflozin 10 mg once daily. The patient was discharged wearing a LifeVest with instructions for strict adherence to medications.

**Figure 4:**
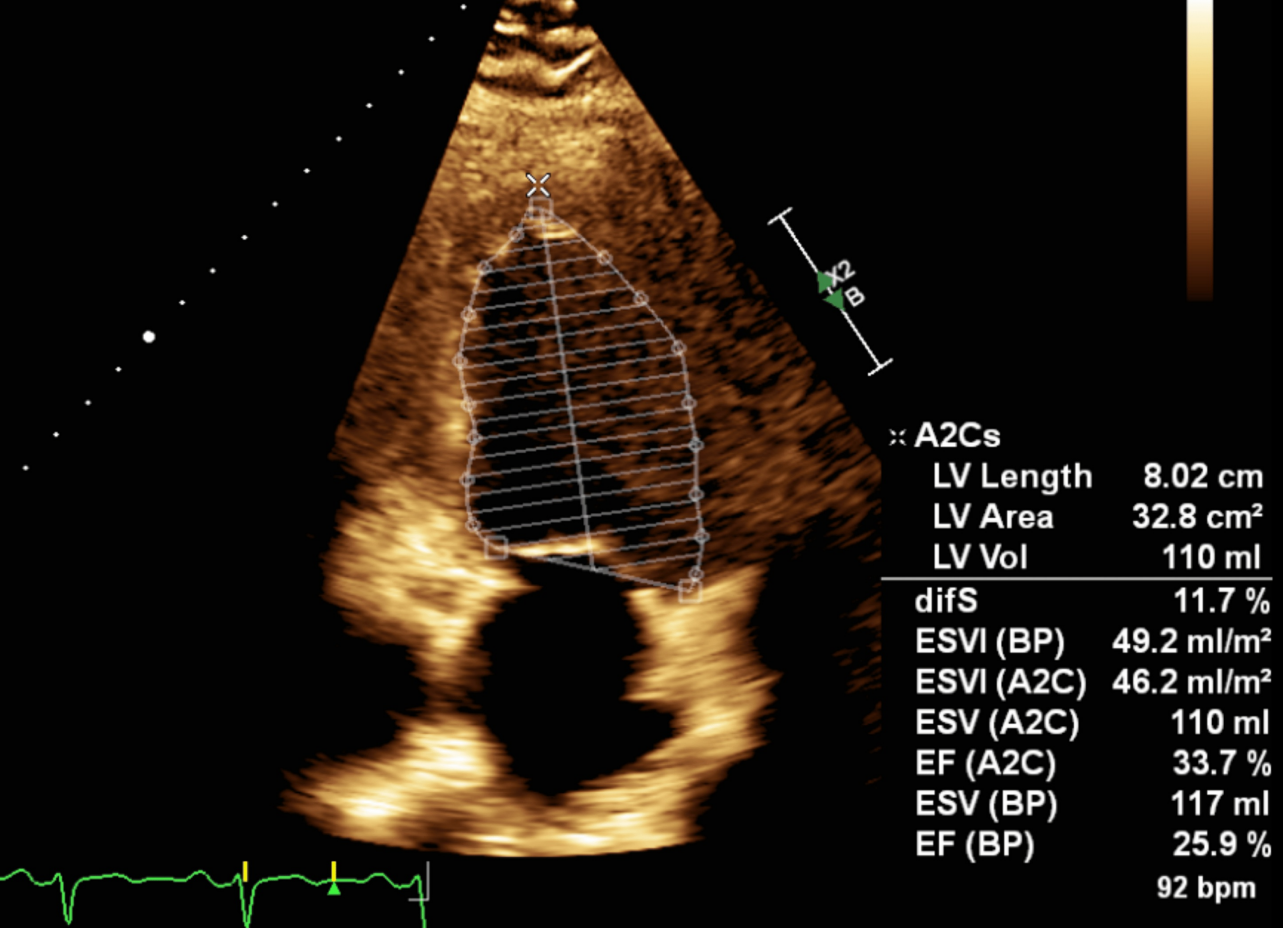
Transthoracic echocardiography (TTE) Apical two-chamber (A2C) view showing left ventricular (LV) volume assessment using Simpson's biplane (BP) method of discs. Measurements demonstrate reduced left ventricular ejection fraction (EF) of 30-35% with increased end-systolic volume (ESV) and end-systolic volume index (ESVI), consistent with impaired LV systolic function following acute myocardial infarction.

**Figure 5:**
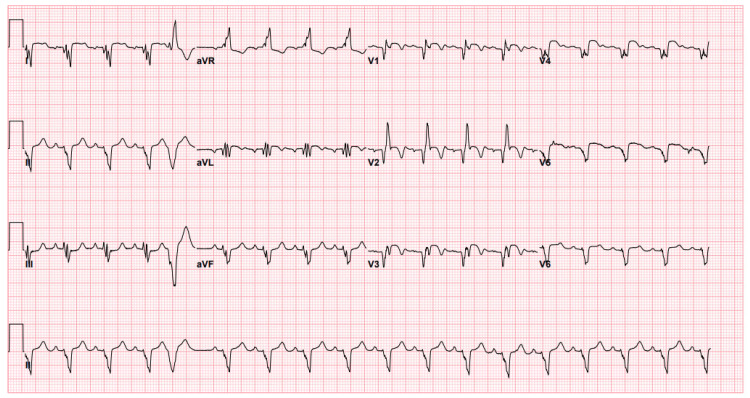
Electrocardiogram on second presentation At the time of the second presentation electrocardiogram demonstrated sinus tachycardia with marked ST-segment elevations in anterolateral leads with reciprocal ST depressions in inferior leads.

**Figure 6:**
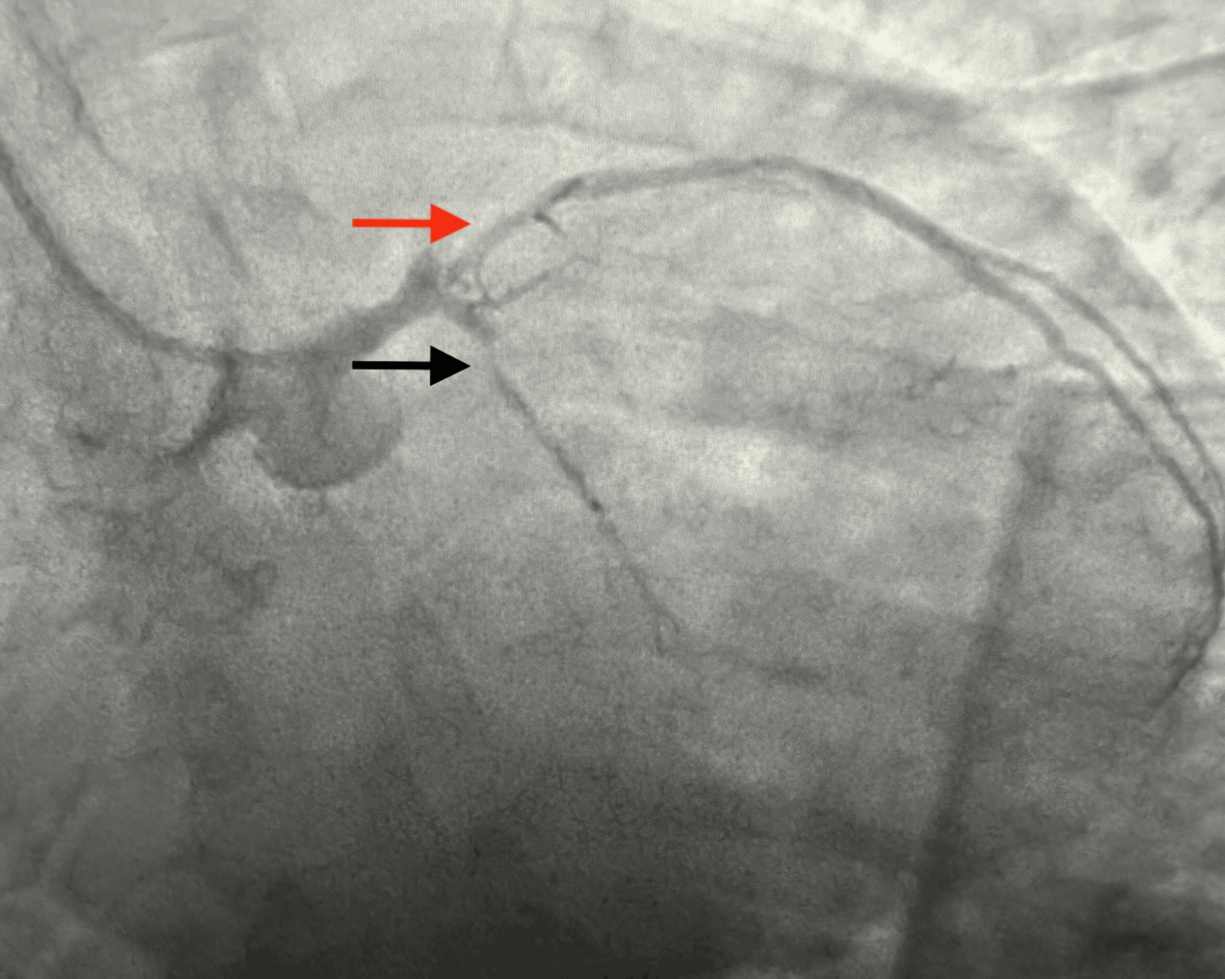
Left heart catheterization on second presentation Coronary angiography obtained during the patient's second presentation with acute ST-segment elevation myocardial infarction demonstrates acute thrombosis involving the previously placed stents in the left anterior descending (LAD) artery (red arrow) and obtuse marginal (OM) branch of the left circumflex artery (black arrow). The angiographic image shows abrupt luminal occlusion with markedly reduced distal coronary flow consistent with early multivessel stent thrombosis.

Within 24 hours of discharge, the patient returned to the ED with recurrent severe (9/10) chest pain that started two hours ago. ECG on arrival demonstrated significant ST-segment elevation in the anterior leads, consistent with an acute anterior ST-segment elevation myocardial infarction (STEMI, Figure 5). He was emergently taken back to the cardiac catheterization lab, where angiography revealed acute thrombosis and occlusion of the previously placed LAD and OM stents (Figure 6). Immediate PCI was performed with anticoagulation using intravenous heparin, and activated clotting time was maintained above 250 seconds. A double bolus of eptifibatide followed by infusion was administered for intensified antiplatelet therapy. Balloon angioplasty of the proximal LAD restored distal flow. Intravascular ultrasound (IVUS) was subsequently performed to further evaluate the previously placed stent and vessel dimensions. IVUS demonstrated a reference vessel diameter of approximately 4.5 mm with areas of relatively suboptimal stent expansion within the previously deployed stent. These findings suggested a potential mechanical factor that may have contributed to thrombus formation.

**Figure 7:**
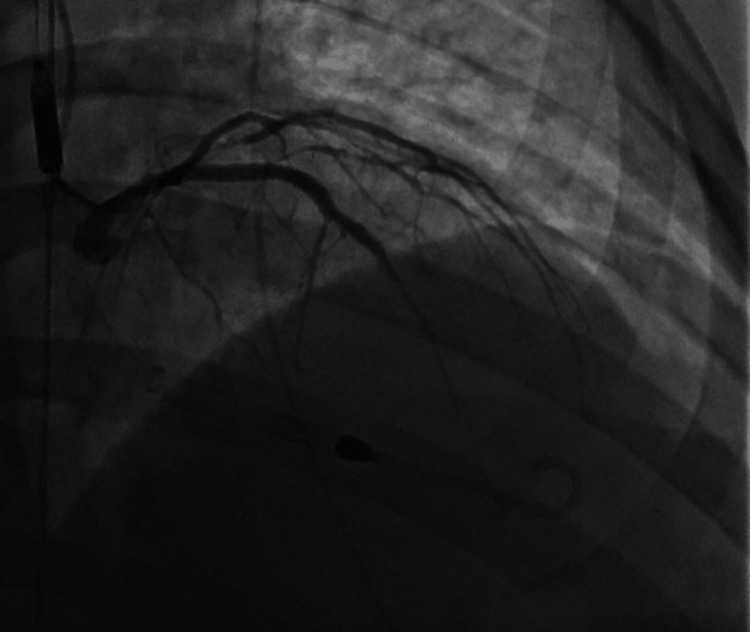
Left heart catheterization Follow-up coronary angiography after intravascular ultrasound-guided repeat percutaneous coronary intervention demonstrates restoration of coronary patency with improved distal flow in the left anterior descending and obtuse marginal arteries. Mechanical circulatory support with an Impella CP^®^ device was initiated due to markedly elevated left ventricular end-diastolic pressure and hemodynamic instability. The device is visualized within the left ventricle, providing temporary circulatory support during high-risk revascularization.

Based on these findings, high-pressure post-dilation was performed using 3.5 mm and 4.0 mm non-compliant balloons. An additional 3.0×38 mm drug-eluting stent was deployed from proximal to mid LAD to optimize the treated segment. Final angiography demonstrated TIMI-3 flow with a good angiographic result and improved stent expansion. Due to significantly elevated left ventricular end-diastolic pressure (LVEDP) approximating 40 mmHg, mechanical circulatory support was initiated with placement of an Impella CP^®^ device via the right femoral artery (Figure 7).

Post-procedure, the patient was treated with intravenous eptifibatide infusion for six hours and systemic anticoagulation with heparin per Impella CP^®^ protocol. A follow-up angiogram performed 24 hours later confirmed stent patency, and the Impella CP^®^ device was subsequently removed. Vital signs before removing the Impella CP^®^ device included blood pressure of 122/93 mmHg, heart rate of 98/min, and temperature of 99.8°F. GDMT was resumed, blood glucose was optimized, and the patient was closely observed for complications during the subsequent days following the procedure. During hospitalization, he was transitioned to ticagrelor as part of dual antiplatelet therapy in combination with aspirin. However, due to cost considerations and lack of outpatient coverage, the patient was discharged on clopidogrel instead of ticagrelor. He remained hemodynamically stable and symptom-free, following which he was discharged home with the instruction to take medications as prescribed and close follow-up with cardiology and primary care physician.

## Discussion

Early stent thrombosis remains a rare but life-threatening complication following PCI. Contemporary studies report an incidence of approximately 0.5-1% with modern drug-eluting stents despite advances in stent design and antiplatelet therapy [6]. The present case illustrates a rare presentation of recurrent acute coronary syndrome due to early multivessel stent thrombosis occurring within 24 hours of discharge. 

Diabetes mellitus and thrombosis risk

Our patient's newly diagnosed diabetes (HbA1C 9.9%) was a likely contributor. Diabetes is associated with platelet hyperactivity, impaired fibrinolysis, and endothelial dysfunction, all of which can increase the risk of stent thrombosis [7]. This highlights the importance of aggressive glycemic control as an integral component of secondary cardiovascular prevention, beyond standard measures. While the conventional secondary prevention strategies, such as dual antiplatelet therapy (DUAT), statins, beta blockers, and renin-angiotensin system inhibition, remain the cornerstone of care, they may be insufficient in patients with poorly controlled diabetes. Early recognition and intensive management of diabetes, including the use of insulin or sodium-glucose cotransporter-2 (SGLT2) inhibitors or glucagon-like peptide 1 (GLP-1) receptor agonists, should be considered essential.

Reduced LVEF as a compounding factor

The patient's new onset heart failure with reduced ejection fraction (EF 30-35%) further compounded the risk of thrombotic stenosis. Heart failure with reduced ejection fraction is associated with systemic inflammation and impaired coronary flow reserve, further predisposing to adverse outcomes [8]. Furthermore, reduced LVEF increases hemodynamic vulnerability, meaning even short episodes of vessel occlusion can result in significant clinical deterioration [9].

Role of IVUS in re-intervention

In the present case, IVUS was performed during the repeat intervention to further evaluate the previously treated segments and vessel dimensions. IVUS demonstrated a relatively large reference vessel diameter of approximately 4.5 mm with areas of less-than-optimal stent expansion within the previously deployed stent. While the angiographic appearance during the index PCI was acceptable, the IVUS findings suggested that relative stent underexpansion within a larger vessel segment may have been a potential contributing factor to thrombus formation. Intracoronary imaging modalities such as IVUS and optical coherence tomography (OCT) have become increasingly important in the evaluation and management of stent-related complications. Compared with angiography alone, IVUS allows more accurate assessment of vessel size, plaque morphology, and stent expansion, which may help identify mechanical factors contributing to stent thrombosis [10]. In this case, IVUS findings guided further optimization of the treated segment with high-pressure balloon dilation and additional stent deployment, resulting in restoration of TIMI-3 coronary flow.

Mechanical circulatory support

Another notable aspect of this case was the patient's hemodynamic instability at the time of the second presentation. On arrival to the emergency department, the patient was hypotensive and tachycardic, with oxygen saturation of 92% that subsequently declined into the 80% range, requiring bilevel positive airway pressure (BiPAP) support. During the procedure, LVEDP was measured at 40 mmHg, indicating markedly elevated filling pressures and compromised cardiac function. Given the combination of elevated LVEDP, ongoing ischemia from multivessel stent thrombosis, and concern for progressive hemodynamic compromise, mechanical circulatory support with an Impella CP^®^ device was selected. Although the intra-aortic balloon pump (IABP) is commonly used for circulatory support in acute coronary syndromes, Impella CP^®^ provides more robust hemodynamic assistance by actively unloading the left ventricle and directly pumping blood into the ascending aorta. This mechanism leads to greater augmentation of cardiac output and more effective reduction in left ventricular end-diastolic pressure compared with IABP [11,12]. In clinical scenarios where significant ventricular unloading is required, such as markedly elevated LVEDP, ongoing myocardial ischemia, or high-risk PCI, Impella CP^®^ may offer superior hemodynamic support compared with IABP, which primarily functions through diastolic augmentation and afterload reduction [11,13]. Therefore, in this patient, the markedly elevated LVEDP and concern for worsening hemodynamic instability during repeat PCI favored the use of Impella CP^®^ to provide more comprehensive ventricular support.

Dual antiplatelet therapy considerations

Despite appropriate initiation of DAPT and adherence to the medications, our patient developed early stent thrombosis, highlighting the challenges of optimizing antiplatelet strategies in high-risk individuals. Clopidogrel remains a commonly prescribed agent given its availability and cost-effectiveness; however, factors such as cytochrome P450 2C19 enzyme (CYP2C19)genetic polymorphisms and high platelet reactivity can contribute to reduced efficacy in certain patients [14]. While routine platelet function or genetic testing is not universally recommended, these tools may be considered in selected patients to refine therapy. Given the concern for potential variability in response to clopidogrel, in our patient, outpatient platelet function testing was recommended to evaluate platelet inhibition. However, the test was not completed due to insurance-related barriers.

## Conclusions

This case highlights the devastating consequences of early stent thrombosis, manifesting as rapid progression from NSTEMI to STEMI despite dual antiplatelet therapy. Multiple factors, including uncontrolled diabetes, impaired left ventricular function, mechanical factors, and potential clopidogrel non-responsiveness, likely contributed to a heightened prothrombotic state. Prompt recognition, intravascular imaging-guided revascularization, and early deployment of Impella CP^®^ support were pivotal in stabilizing the patient and facilitating recovery. For high-risk individuals, physicians should remain vigilant for early recurrence, emphasizing personalized antiplatelet therapies, precise procedural optimization, and close post-PCI surveillance to minimize this life-threatening complication.
